# Evolutionary engineered *Candida intermedia* exhibits improved xylose utilization and robustness to lignocellulose-derived inhibitors and ethanol

**DOI:** 10.1007/s00253-018-9528-x

**Published:** 2018-11-29

**Authors:** Antonio D. Moreno, Antonella Carbone, Rosita Pavone, Lisbeth Olsson, Cecilia Geijer

**Affiliations:** 1 Department of Biology and Biological Engineering, Division of Industrial Biotechnology, Chalmers University of Technology, 41296 Gothenburg, Sweden; 2 Department of Energy, Biofuels Unit, CIEMAT, Madrid, Spain

**Keywords:** Non-conventional yeast, Xylose fermentation, Microbial robustness, Lignocellulosic bioethanol

## Abstract

The development of robust microorganisms that can efficiently ferment both glucose and xylose represents one of the major challenges in achieving a cost-effective lignocellulosic bioethanol production. *Candida intermedia* is a non-conventional, xylose-utilizing yeast species with a high-capacity xylose transport system. The natural ability of *C. intermedia* to produce ethanol from xylose makes it attractive as a non-GMO alternative for lignocellulosic biomass conversion in biorefineries. We have evaluated the fermentation capacity and the tolerance to lignocellulose-derived inhibitors and the end product, ethanol, of the *C. intermedia* strain CBS 141442 isolated from steam-exploded wheat straw hydrolysate. In a mixed sugar fermentation medium, *C. intermedia* CBS 141442 co-fermented glucose and xylose, although with a preference for glucose over xylose. The strain was clearly more sensitive to inhibitors and ethanol when consuming xylose than glucose. *C. intermedia* CBS 141442 was also subjected to evolutionary engineering with the aim of increasing its tolerance to inhibitors and ethanol, and thus improving its fermentation capacity under harsh conditions. The resulting evolved population was able to ferment a 50% (*v*/*v*) steam-exploded wheat straw hydrolysate (which was completely inhibitory to the parental strain), improving the sugar consumption and the final ethanol concentration. The evolved population also exhibited a better tolerance to ethanol when growing in a xylose medium supplemented with 35.5 g/L ethanol. These results highlight the potential of *C. intermedia* CBS 141442 to become a robust yeast for the conversion of lignocellulose to ethanol.

**Electronic supplementary material:**

The online version of this article (10.1007/s00253-018-9528-x) contains supplementary material, which is available to authorized users.

## Introduction

Concerns about global warming and uncertainties in the future energy supply are important driving forces behind the development and implementation of a sustainable bio-based economy. Biorefineries will be used in the future to transform biomass into a wide range of products, including fuels and other energy forms, high value-added chemicals, and other materials, similar to current petroleum-based refineries (Olsson and Saddler 2013). Lignocellulosic biomass is the most abundant form of organic matter in nature (Sánchez and Cardona 2008) and is hence considered the foremost source of raw material for use in biorefineries. Among biomass-derived products, lignocellulosic bioethanol represents an important bulk chemical for the replacement of fossil fuels in the short to medium term. Although significant progress has recently been made towards the commercialization of lignocellulosic bioethanol, several bottlenecks must be resolved to achieve economic and sustainable conversion processes (Balan 2014; Gubicza et al. 2016).

Lignocellulose is composed of three main polymers: cellulose, hemicelluloses, and lignin, linked by covalent and non-covalent bonds to form a complex matrix. Biochemical conversion of lignocellulose includes three major steps: (1) pretreatment, to open up the inherent recalcitrant structure, (2) enzymatic hydrolysis, for the saccharification of carbohydrate polymers, and (3) microbial fermentation, to transform the hydrolyzed sugars into useful products such as ethanol. The pretreatment step is usually performed under harsh conditions (such as high temperatures and pressures and/or low/high pH), which leads to extensive biomass degradation, generating various lignocellulose-derived compounds such as aliphatic acids, furan derivatives, and several phenolic compounds (Alvira et al. 2010). These compounds inhibit hydrolytic enzymes as well as fermentative microorganisms, thereby decreasing both the overall conversion yields and the microbial fermentation capacity (Taherzadeh and Karimi 2011; Jönsson et al. 2013). For instance, the furan derivatives furfural and 5-hydroxymethylfurfural have a direct impact on fermentative cells by inhibiting glycolytic and/or fermentative enzymes, thus reducing biomass formation and ethanol yields (Horváth et al. 2003). Furthermore, inhibitory compounds have been shown to exert a synergistic action on fermentative microorganisms, which can inhibit their growth, even at low concentrations (Oliva et al. 2004; Alvira et al. 2013). Another relevant stress factor affecting fermentation is the end product, ethanol, as it impedes cell growth and reduces viability, thus limiting the fermentation productivity and final conversion yields (Stanley et al. 2010).

Most fermentative microorganisms are capable of removing and/or tolerating inhibitory compounds (Ask et al. 2013; Lindberg et al. 2013; Adeboye et al. 2017). This natural robustness can be improved by subjecting the microorganism to evolutionary engineering (Sauer 2001), by exposing the cells to sublethal inhibitory concentrations of a specific stressor to promote enrichment of cells with a selective advantage at the expense of the initially dominant cells. The advantages of using evolutionary engineering to increase microbial robustness are (1) no detailed knowledge of the microorganism is necessary, (2) resistance to multiple stress factors may be promoted at the same time, and (3) no external genetic modifications are introduced, thus preserving the non-GMO classification of the microorganism (Sauer 2001; Koppram et al. 2012).

*Saccharomyces cerevisiae* is currently the most commonly used fermentative microorganism in the starch-based bioethanol industry due to its superior fermentation capacity of hexose sugars, particularly glucose. Moreover, in comparison with most other microorganisms characterized to date, *S. cerevisiae* exhibits a high tolerance to ethanol as well as lignocellulose-derived inhibitors (Piskur et al. 2006; Stanley et al. 2010; Parawira and Tekere 2011; Koppram et al. 2014). However, the major disadvantage of using *S. cerevisiae* strains to produce bioethanol from lignocellulosic materials is its inability to ferment pentoses such as D-xylose and L-arabinose (Sun and Cheng 2002; Hahn-Hägerdal et al. 2007). As xylose is the second most prevalent sugar monomer after glucose in lignocellulosic hydrolysates, and hence a highly important substrate, extensive research efforts have been made to introduce heterologous genes for xylose metabolism into *S. cerevisiae* (Moyses et al. 2016). These metabolic engineering approaches are often followed by evolutionary engineering and/or inverse metabolic engineering to optimize the xylose uptake and fermentation capacity. Although considerable progress has been achieved, engineered strains still suffer from inefficient xylose uptake and sequential fermentation of glucose and xylose (Subtil and Boles 2012). Furthermore, inefficient cofactor recycling during the catalysis of the NADPH-preferring xylose reductase and the NAD^+^-dependent xylitol dehydrogenase enzymes results in the accumulation of xylitol as a by-product, thus reducing the overall yield of ethanol on xylose (Jeffries and Jin 2004). Native xylose-fermenting yeasts, including species of the genera *Scheffersomyces* (*Scheffersomyces stipitis* and *Scheffersomyces shehatae*), *Spathaspora* (*Spathaspora passalidarum*), *Pachysolen* (*Pachysolen tannophilus*), and *Candida* (*Candida tropicalis* and *Candida tenuis*), can be considered as alternatives to these genetically modified *S. cerevisiae* strains (Sánchez et al. 2002; Gárdonyi et al. 2003; Su et al. 2015).

The yeast *Candida intermedia* is also an interesting pentose-fermenting microorganism since it exhibits similar specific growth rates in glucose and xylose (Gárdonyi et al. 2003), expresses potent xylose transporters (Leandro et al. 2006), and has been shown to ferment glucose and xylose at high concentrations (Saito et al. 2017). Furthermore, it harbors multiple isoforms of xylose reductases, one of which has dual cofactor specificity, which may contribute to a better redox balance (Nidetzky et al. 2003). A new strain of *C. intermedia*, CBS 141442, was recently isolated in our lab from the liquid fraction of a steam-pretreated wheat straw hydrolysate (Moreno et al. 2017). The aim of this study was to determine the capacity of CBS 141442 to ferment glucose and xylose and its tolerance to lignocellulose-derived inhibitors and ethanol. Moreover, the strain was subjected to evolutionary engineering to improve its robustness and its ability to ferment xylose under limiting conditions.

## Materials and methods

### Lignocellulosic hydrolysate

The liquid fraction (hydrolysate) collected from an acid-catalyzed steam-exploded wheat straw was used as a source of lignocellulose-derived inhibitors. The hydrolysate was recovered by vacuum filtration of the whole pretreated slurry, which was kindly provided by SEKAB E Technology (Örnsköldsvik, Sweden). Selected pretreatment conditions were similar to those reported as “mild pretreatment” by Nielsen et al. (2016). The chemical composition of the collected hydrolysate was analyzed as described below and is given in Table 1.

**Table 1:** Chemical composition of the lignocellulosic hydrolysate obtained after acid-catalyzed steam explosion pretreatment of wheat straw

Compound	Concentration (g/L)
Sugars	
*Glucose*	6.0 ± 0.5
*Xylose*	34.1 ± 1.0
*Arabinose*	5.5 ± 0.3
*Galactose*	1.9 ± 0.2
Inhibitory compounds	
*Acetic acid*	5.7 ± 0.5
*Formic acid*	0.5 ± 0.2
*Furfural*	3.9 ± 0.4
*5-HMF*	0.8 ± 0.1
pH	3.1
*5-HMF* 5-hydroxymethylfurfural	

The hydrolysate was divided into two batches. One batch was supplemented with glucose, up to 20 g/L, and the pH adjusted to 5, and was used for evolutionary engineering and cell pre-adaptation during inoculum preparation. The second batch was first diluted with water to a final concentration of 30–50% (*v*/*v*), supplemented with (NH_4_)_2_PO_4_ (0.5 g/L), glucose (up to 10 g/L) and xylose (up to 20–30 g/L), and the pH adjusted to 5.5, and used in fermentation assays. Both batches were stored at 4 °C prior to use.

### Microorganisms and medium composition

The in-house isolated yeast *C. intermedia* CBS 141442 (haploid strain) was used as the parental strain in the present work (Moreno et al. 2017). This strain was subjected to evolutionary engineering as described below, resulting in two evolved populations: *C. intermedia* EVO 1 and *C. intermedia* EVO 2. Cells were stored at − 80 °C in 20% (*v*/*v*) glycerol prior to inoculation.

*C. intermedia* cells were grown in liquid minimal mineral medium (MM) (7.5 g/L (NH_4_)_2_SO_4_, 3.5 g/L KH_2_PO_4_, 0.75 g/L MgSO_4_·7H_2_O, 2 mL/L trace metal solution, and 1 mL/L vitamin solution) (Verduyn et al. 1990), or rich medium (YP) (10 g/L yeast extract and 20 g/L peptone), both were supplemented with 20 g/L glucose (MMD, YPD), 20 g/L xylose (MMX, YPX), or 10 or 40 g/L glucose and 20 g/L xylose (MM10G20X; MM40G20X).

### Random mutagenesis and sequential evolutionary engineering

**Fig. 1:**
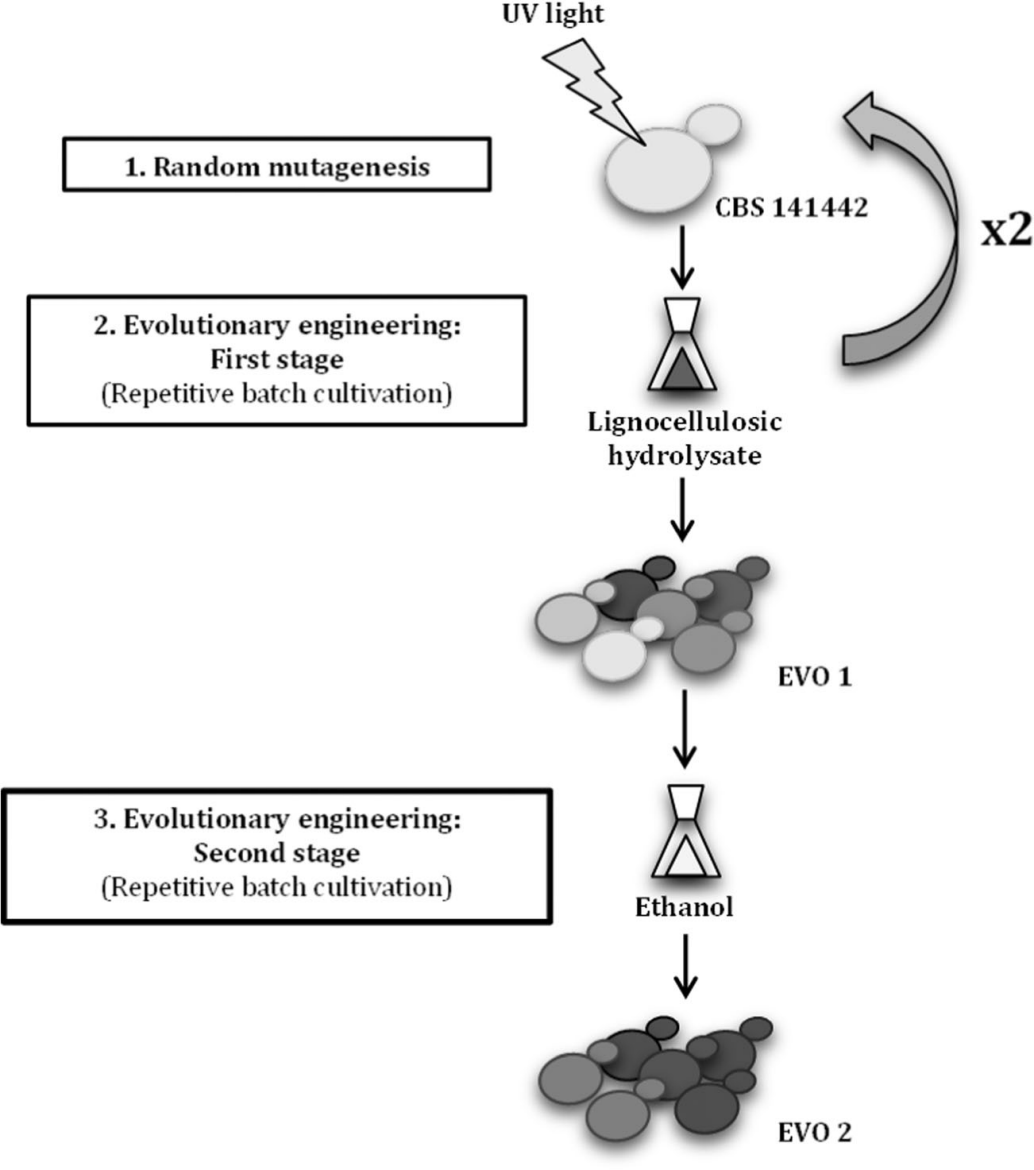
Schematic representation of the evolutionary engineering of *C. intermedia* CBS 141442. The evolved population EVO 1 was obtained after 2 cycles of random mutagenesis with UV light and short-term adaptation in the presence of lignocellulose-derived inhibitors (5–30% (*v*/*v*) wheat straw hydrolysate). This intermediate population was subsequently subjected to short-term adaptation in the presence of 31.6 g/L ethanol, resulting in the final evolved population EVO 2

*C. intermedia* 141442 was subjected to sequential evolutionary engineering in the presence of lignocellulose-derived inhibitors and ethanol (Fig. 1). Prior to evolutionary engineering, *C. intermedia* cells were randomly mutagenized using UV light. Cells from MM cultures (100 μL, OD_600_ = 1) were spread on MM agar plates and placed upside-down with lids removed on a UV-transilluminator (UVP, Cambridge, UK). High-intensity irradiation capacity at a wavelength of 302 nm (UVB), which is known to induce DNA mutations (Armstrong and Kunz 1990), was used for 20, 40, and 60 s according to preliminary data showing low, mid, and mid-high % kill. Non-treated and UV-treated cells were then pooled together to create a start population with a large genetic variability, inoculated into a 100-mL flask containing 50 mL selective medium at 5% (*v*/*v*) hydrolysate concentration (YP supplemented with 20 g/L xylose and 5% (*v*/*v*) hydrolysate), and incubated in an orbital shaker at 30 °C and 100–150 rpm.

The first stage of the evolutionary engineering process was performed at increased concentrations of lignocellulose-derived inhibitors, and cells were grown in repetitive batch cultures with selective medium at 5–15% (*v*/*v*) hydrolysate concentration. Briefly, the concentration of hydrolysate was increased from 5 to 7.5%, 10%, 12.5%, and 15% (*v*/*v*), after observing an increase in specific growth rate in the corresponding selective medium. Each subculturing round started with an initial OD_600nm_ value of 0.05. The cells capable of growing in selective medium at 15% (*v*/*v*) were then subjected to a second round of UV mutagenesis, following the same procedure as described above. The pool of UV-treated cells was then inoculated into 50 mL selective medium at 20% (*v*/*v*) hydrolysate concentration, continuing the evolution rounds until cells were capable of growing in selective medium at 30% (*v*/*v*) hydrolysate concentration (a 2-fold increase in the hydrolysate concentration tolerated by the parental strain). The intermediate population *C. intermedia* EVO 1 was obtained from this first stage of the evolution process.

A similar subculturing procedure was used in the second stage of the process to further evolve *C. intermedia* EVO 1 using ethanol as the selective pressure. Repetitive batch cultures were performed in YPX medium supplemented with 31.6 g/L ethanol. Each new subculturing round started with an initial OD_600nm_ value of 0.1, since a higher initial biomass content was needed to overcome growth inhibition. To avoid ethanol oxidation by *C. intermedia* cells as evolutionary strategy, shaking was maintained at 100 rpm during the whole evolution process. Cells were grown under these conditions until an increase in the specific growth rate was observed (about 12 rounds). This second stage of evolutionary engineering resulted in the final evolved population *C. intermedia* EVO 2.

To monitor the cultures for contaminating wild yeasts, cells were sampled both during the evolutionary engineering experiments and from the end populations EVO 1 and EVO 2. DNA was extracted and the ITS region was amplified with PCR using the ITS1 and ITS4 primers (White et al. 1990), and the species of origin was determined by sequencing and/or estimating the size of the PCR product.

Whenever the evolved populations EVO 1 and EVO 2 were used to inoculate starter cultures for fermentation tests, cells were diluted about 1000 times to ensure that they grew for 10–11 generations in non-selective medium before harvest and reinoculated in the fermentation medium. This way, stable genetic changes rather than short-term adaptation mechanisms are the expected causes for the improved tolerance of the evolved populations.

### Fermentation of synthetic medium and lignocellulosic hydrolysate

The fermentation performance of *C. intermedia* was evaluated in the presence and absence of lignocellulose-derived inhibitors. In the absence of inhibitory compounds, MM10G20X and MM40G20X were used as fermentation media, while 30%, 40%, or 50% (*v*/*v*) hydrolysates were used to evaluate the inhibitory tolerance. In all cases, fermentation was performed at 30 °C and 150 rpm in 100-mL shake flasks, with 30 mL of the appropriate fermentation medium, using an inoculum concentration of 3 g/L cell dry weight. Fermentation assays were carried out in duplicate or triplicate under reduced oxygen concentration conditions by employing an airlock system that allows CO_2_ outflow and prevents O_2_ inflow. Samples were withdrawn periodically for analytical purposes. The results are presented as the average values and standard deviations.

Prior to inoculation, precultivated cells were obtained by incubation for 24 h at 30 °C and 150 rpm in 500-mL baffled flasks containing 100 mL MM10G20X. Furthermore, when 30–50% (*v*/*v*) hydrolysate was used as fermentation medium, a pre-adaptation phase was included during preculture, since this has been shown to be crucial for xylose fermentation (Nielsen et al. 2015). Thus, after 16–18 h of cell culture (OD_600nm_ = 2–3), lignocellulosic hydrolysate (that supplemented with 20 g/L glucose and a pH of 5) was added to a final concentration of 15% (*v*/*v*). The cultures were then further incubated under the same conditions (30 °C and 150 rpm) for 8 h. Active cells were harvested by centrifugation at 5000*g* at room temperature for 5 min. The supernatant was discarded, and the cell pellet was washed once with sterile water. The cell pellet was then weighed to obtain the desired inoculum size.

### Cell growth in the presence of ethanol

The tolerance of *C. intermedia* to ethanol was evaluated in YPD and YPX media supplemented with 0–39.5 g/L ethanol. For preculture, cells growing in the exponential phase, in YPD or YPX, were transferred to 50 mL of the corresponding test medium to a final OD_600nm_ of 0.1. The cultures were then incubated in an orbital shaker at 30 °C at 150 rpm for 48 h. Samples were withdrawn periodically to monitor cell growth in terms of OD_600nm_. To evaluate the effect of ethanol on yeast growth, kinetic parameters related to ethanol inhibition were estimated using Luong’s eq. (1) (Luong 1985):

1 $$ \frac{\mu_e}{\mu_0}=1-{\left(\frac{P}{P_{\mathrm{max}}}\right)}^{\alpha } $$

where μ_e_ and *μ*_*0*_ are the maximum specific growth rates (h^−1^) in the presence and absence of ethanol, respectively; *P* is the ethanol concentration (g/L); *P*_*max*_ is the critical ethanol concentration (g/L) above which there is no growth; and *α* is the ethanol tolerance index, which describes the type of inhibition (*α* = 1 linear relationship; *α* < 1 hyperbolic relationship; *α* > 1 parabolic relationship). Values of *P*_*max*_ and *α* were obtained by plotting *Ln* (*1–μ*_*e*_*/μ*_*0*_) versus *Ln* (*P*).

### Analytical methods

The concentrations of glucose, xylose, xylitol, glycerol, acetate, ethanol, 5-hydroxymethylfurfural (5-HMF), and furfural were determined using high-performance liquid chromatography (Dionex-Thermo Fisher Scientific, Sunnyvale, CA, USA), with a Rezex ROA-organic acid H^+^ column (Phenomenex, Torrance, CA, USA). The operating temperature was 80 °C and the flow rate of the mobile phase (5 mM H_2_SO_4_) was 0.8 mL/min. Compounds were identified using a refractive index detector at 35 °C (Dionex RI-101) and a UV detector at 210 nm (UltiMate™ 3000 VWD), both from Dionex-Thermo Fisher Scientific (Sunnyvale, CA, USA).

Cell growth was monitored spectrophotometrically (Genesys 20 Spectrophotometer; Thermo Fisher Scientific, Waltham, MA, USA) by measuring the absorbance at 600 nm. Maximum specific growth rates (μ_max_ (h^−1^)) were estimated when cells were growing in the exponential phase using linear regression analysis, by plotting *Ln* (*OD*_*600nm*_) versus *t* (h).

The xylose consumed (*C*_*X*_ (%)) Eq. (2), xylose consumption rates (*Q*_*X*_ (g/L h)) Eq. (3) and ethanol yield (*YE* (g/g)) Eq. (4) were estimated as follows:

2 $$ {C}_x\left(\%\right)=\frac{\left( xylos{e}_i- xylos{e}_f\right)\times 100}{xylos{e}_i} $$

3 $$ {Q}_x\left(g/ Lh\right)=\frac{\left( xylos{e}_i- xylos{e}_f\right)}{\left({t}_f-{t}_i\right)} $$

4 $$ {Y}_E\left(g/g\right)=\frac{ethano{l}_f}{\left( sugar{s}_i- sugar{s}_f\right)} $$

where the subscripts _i_ and _f_ denote the initial and final concentrations in g/L, respectively.

## Results

### Fermentation performance of *C. intermedia* CBS 141442 in the absence of inhibitory compounds

Efficient conversion of both glucose and xylose is required for cost-effective lignocellulosic bioethanol production. To determine the suitability of *C. intermedia* CBS 141442 as a cell factory for this purpose, its fermentative performance was evaluated in the absence of inhibitory compounds. *C. intermedia* is a Crabtree-negative yeast, and thus, the fermentation tests were conducted under low oxygen conditions, as this is a prerequisite for fermentation in these yeasts (Rizzi et al. 1989). We also evaluated the fermentation capacity in different initial glucose concentrations, as the glucose content of lignocellulosic hydrolysates is dependent on whether cellulose is hydrolyzed and collected with the corresponding solubilized hemicelluloses or not (Olsson et al. 2005). Hence, minimal medium supplemented with 20 g/L xylose and either 10 or 40 g/L glucose (MM10G20X and MM40G20X) was used.

**Fig. 2:**
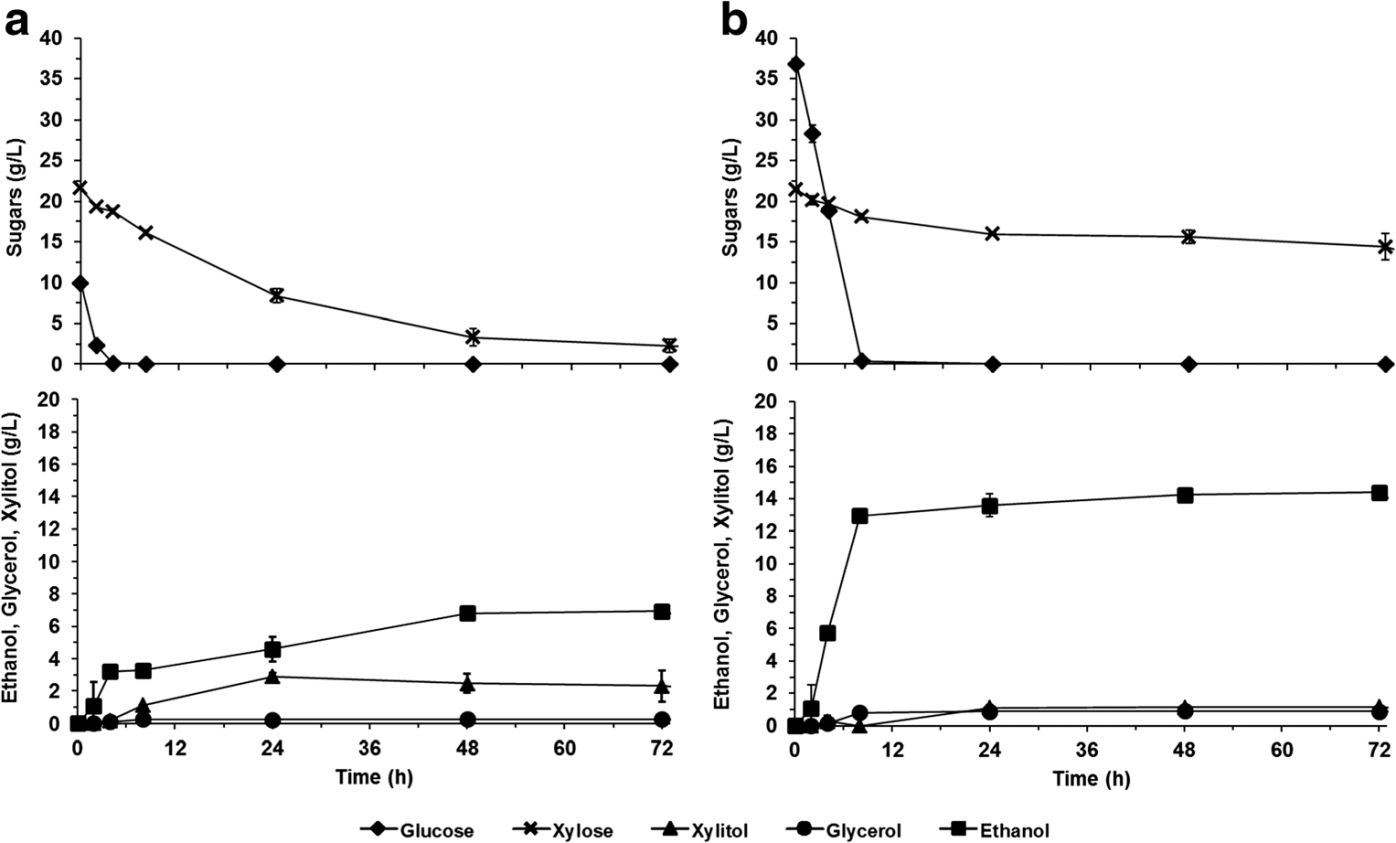
Fermentation of mineral media with an initial xylose concentration of 20 g/L and a glucose concentration of **a** 10 g/L (MM10G20X) or **b** 40 g/L (MM40G20X) by the parent *C. intermedia* strain CBS 141442

*C. intermedia* strain CBS 141442 was capable of simultaneous utilization of glucose and xylose during fermentation of MM10G20X and MM40G20X, producing maximum ethanol concentrations of 6.9 g/L and 14.4 g/L after 72 h, respectively (Fig. 2, Table 2). Based on the amounts of the sugars consumed, these ethanol concentrations correspond to yields of 0.24 g/g and 0.33 g/g, which in turn represent 47% and 65% of the theoretical amounts of ethanol that could be produced (considering a theoretical ethanol yield of 0.51 g/g from both glucose and xylose). In addition to ethanol, 2.9 g/L xylitol was produced when using MM10G20X, while 1.2 g/L xylitol and 1 g/L glycerol were obtained with MM40G20X (Fig. 2, Table 2). Under these conditions, glucose was always consumed at a higher rate than xylose, showing complete depletion within the first 8 h (Fig. 2). Moreover, different xylose consumption profiles were observed, depending on the initial glucose concentration. With an initial glucose concentration of 10 g/L (MM10G20X), a xylose consumption rate of 0.53 g/L h was reached within the first 24 h, sustained xylose consumption was observed throughout the whole fermentation process, and the initial xylose concentration was reduced by 90% after 72 h. When the initial glucose concentration was increased to 40 g/L (MM40G20X), the initial xylose consumption rate decreased by 2.5-fold (0.21 g/L h), and almost, no xylose consumption was observed after 24 h (over 67% of the initial xylose concentration remained in the fermentation medium after 72 h). It is important to note that these experiments were performed in shake flasks without monitoring the oxygen concentration. Thus, a higher initial glucose concentration could lead to increased oxygen consumption, reaching concentrations that are limiting for xylose assimilation (Rizzi et al. 1989). Furthermore, after glucose depletion, the ethanol tolerance of *C. intermedia* CBS 141442 might be not sufficient to allow continued xylose fermentation (Harner et al. 2015). Nevertheless, these results show that *C. intermedia* CBS 141442 can co-ferment glucose and xylose, although it has a preference for glucose.

**Table 2:** Kinetic parameters obtained during the fermentation of mineral media (MM10G20X and MM40G20X) and 30–50% (*v*/*v*) lignocellulosic hydrolysate with *C. intermedia* CBS 141442 and the evolved populations EVO 1 and EVO 2

Strain/population	Fermentation medium	Sugars_i_ (g/L)^a^	Ethanol_max_ (g/L)^b^	C_X_ (g/L)^c^	Q_X_ (g/L h)	Xylitol (g/L)	Y_E_ (g/g)^d^
*C. intermedia* CBS 141442	MM10G20X	31.6 ± 0.3 (10 ± 0.1)	6.9 ± 0.0 (72 h)	19.4 ± 1.1 (89.5%)	0.53 ± 0.04^e^	2.9 ± 0.2	0.24 ± 0.01
*C. intermedia* CBS 141442	MM40G20X	58.3 ± 0.2 (36.8 ± 0.1)	14.4 ± 0.3 (72 h)	7.1 ± 1.7 (32.9%)	0.21 ± 0.01^e^	1.2 ± 0.0	0.33 ± 0.01
*C. intermedia* CBS 141442	30% (v/v) LH	41.2 ± 0.3 (10.6 ± 0.1)	7.4 ± 1.3 (72 h)	16.6 ± 4.4 (54.6%)	0.22 ± 0.06	3.8 ± 0.5	0.27 ± 0.01
*C. intermedia* CBS 141442	40% (v/v) LH	40.2 ± 0.0 (10.7 ± 0.0)	6.4 ± 0.4 (72 h)	11.6 ± 1.6 (38.0%)	0.14 ± 0.03	3.3 ± 0.1	0.29 ± 0.00
*C. intermedia* CBS 141442	50% (v/v) LH	31.4 ± 0.1 (10.4 ± 0.0)	0.0 ± 0.0 (120 h)	0.0 ± 0.0 (0.0%)	0.00 ± 0.00	0.0 ± 0.0	0.00 ± 0.00
*C. intermedia* EVO 1	50% (v/v) LH	34.0 ± 0.2 (12.2 ± 0.0)	7.5 ± 0.3 (120 h)	16.3 ± 1.9 (75.2%)	0.16 ± 0.02	3.8 ± 1.1	0.26 ± 0.03
*C. intermedia* EVO 2	50% (v/v) LH	33.8 ± 0.1 (12.1 ± 0.0)	7.4 ± 0.3 (120 h)	16.0 ± 2.1 (73.9%)	0.16 ± 0.03	3.7 ± 0.5	0.26 ± 0.01

*Sugarsi* total initial glucose and xylose concentration, *Ethanolmax* maximum ethanol concentration, *CX* xylose consumed, *QX* xylose consumption rate, *YE* ethanol yield, *LH* lignocellulosic hydrolysate

^a^Glucose concentration (g/L) is given in brackets

^b^Fermentation time is given in brackets

^c^Percentage of the xylose consumed is given in brackets

^d^Ethanol yield was calculated based on consumed glucose and xylose

^e^QX calculated based on the values within the first 24 h of fermentation. The glycerol concentration remained below 1 g/L in all fermentations

### Effect of lignocellulose-derived inhibitors on growth and fermentation performance of *C. intermedia* CBS 141442

The fermentation performance of *C. intermedia* CBS 141442 was further investigated in the presence of lignocellulose-derived inhibitors by using the liquid fraction collected from steam-exploded wheat straw. Fermentation of wheat straw hydrolysate at 30% and 40% (*v*/*v*) yielded maximum ethanol concentrations of 7.4 g/L and 6.4 g/L, respectively (Table 2). These ethanol concentrations correspond to conversion yields of 0.27 g/g and 0.29 g/g, based on the consumed sugars. Xylitol was also accumulated during the fermentation process, reaching final concentrations of 3.8 g/L (30% (*v*/*v*) hydrolysate) and 3.3 g/L (40% (*v*/*v*) hydrolysate). Despite the similar conversion yields, a lower final ethanol concentration was observed during the fermentation of 40% (*v*/*v*) hydrolysate due to the reduced xylose consumption (from 55 to 38%). A further increase in the hydrolysate concentration from 40 to 50% (*v*/*v*) resulted in complete inhibition of *C. intermedia* CBS 141442, and no sugar consumption or ethanol production was observed within 120 h (Table 2).

Two of the most important lignocellulose-derived inhibitors, furfural and 5-HMF (10 mM), were sequentially converted by *C. intermedia* in the presence of glucose, whereas the yeast failed to convert these inhibitors in the presence of xylose (Supplementary Fig. S1). Thus, the presence of glucose during the initial stages of the fermentation process benefits the conversion of these biomass-derived inhibitors. After glucose depletion, non-converted inhibitory compounds might have a greater influence on the yeast cells, limiting xylose-to-ethanol conversion, thus explaining the results presented in Table 2. Therefore, in order to improve xylose conversion and ethanol production by *C. intermedia*, its tolerance to lignocellulose-derived inhibitors must be enhanced.

### Effect of ethanol on growth of *C. intermedia* CBS 141442

**Fig. 3:**
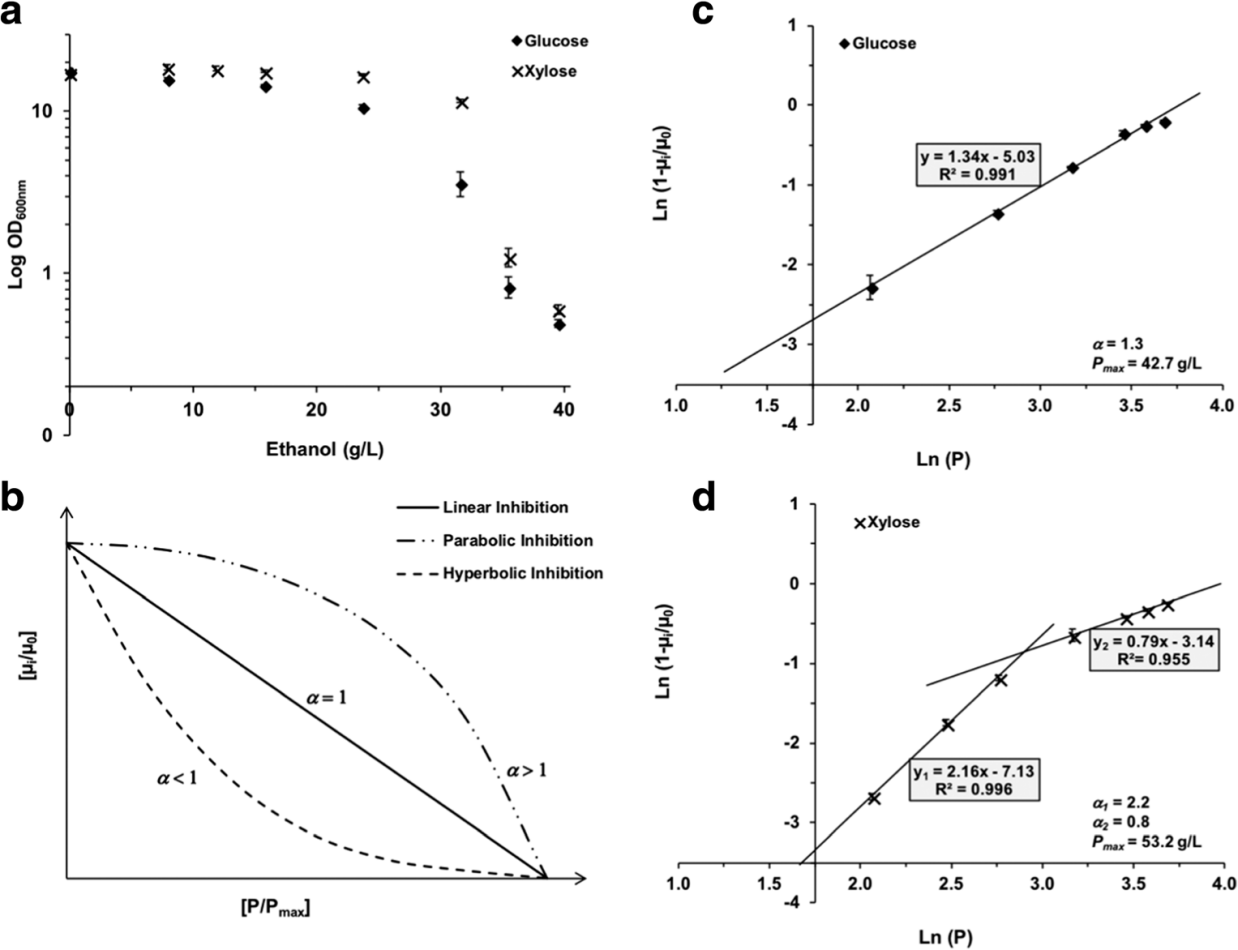
Effect of ethanol on the growth of *C. intermedia* strain CBS 141442 in rich media with glucose (YPD) or xylose (YPX). **a** Biomass formation in terms of OD_600nm_ after 48 h of cultivation. **b** Inhibition mechanisms according to the ethanol tolerance index (*α*). **c** Estimation of *P*_*max*_ and *α* in the glucose-containing medium. **d** Estimation of *P*_*max*_ and *α* in the xylose-containing medium

Ethanol is another important inhibitor of cell growth during the conversion of lignocellulose to ethanol. The tolerance of *C. intermedia* CBS 141442 to different ethanol concentrations was first examined in terms of biomass formation (by measuring OD_600nm_), in rich medium supplemented with glucose or xylose (YPD/YPX medium was used due to difficulties in measuring OD_600nm_ in ethanol-supplemented MMD/MMX medium, since the cells agglomerated). Cells subjected to ethanol stress produce less energy, which in turn affects energy-demanding processes such as growth (Stanley et al. 2010). No differences were observed in terms of biomass formation below an ethanol concentration of 15.8 g/L, regardless of which sugar was present in the medium (Fig. 3a). However, lower biomass concentrations were observed at ethanol concentrations above 23.7 g/L in the presence of glucose (YPD medium) and above 31.6 g/L in the presence of xylose (YPX medium).

To determine the extent to which ethanol inhibits cell growth, kinetic parameters for ethanol inhibition of *C. intermedia* strain CBS 141442 were determined using the kinetic model proposed by Luong (1985). This model establishes the relationship between the specific growth rate (*μ*) and the critical ethanol concentration (*P*_*max*_), which is described by the ethanol tolerance index (*α*) (Fig. 3b). When *α* > 1 (parabolic inhibition), a slow initial decrease in the specific growth rate is followed by a rapid decrease to zero at sublethal inhibition concentrations. In contrast, when *α* < 1 (hyperbolic inhibition), a rapid initial decrease is seen in the specific growth rate followed by a slow decrease to zero as the ethanol concentration is increased. Thus, lower *α* values indicate greater growth inhibition. In the case of *C. intermedia* CBS 141442 cells growing in glucose, *α* and *P*_*max*_ were estimated to be 1.3 and 42.7 g/L, respectively (Fig. 3c). However, cells growing in xylose showed two different inhibition trends, with a turning point at an ethanol concentration of 18.4 g/L (Fig. 3d). At this ethanol concentration, *α* decreased from 2.2 to 0.8 and the critical ethanol concentration (*P*_*max*_) was estimated to be 53.2 g/L. This means that when *C. intermedia* CBS 141442 is growing in the xylose-containing media with ethanol concentrations exceeding 18.4 g/L, the cells are more severely inhibited per unit ethanol than in the corresponding media containing glucose (Fig. 3a). Apart from the lower tolerance to lignocellulosic-derived compounds in the presence of xylose, the lower tolerance to ethanol when this sugar is present may represent a major drawback for the utilization of *C. intermedia* in the ethanol industry.

### Evolutionary engineering of *C. intermedia* CBS 141442

In order to improve xylose conversion and ethanol production by *C. intermedia* CBS 141442, the strain was subjected to evolutionary engineering in two sequential steps to increase its tolerance to lignocellulose-derived compounds (first step) and to ethanol (second step), resulting in the evolved populations EVO 1 and 2 (as described in materials and methods and depicted in Fig. 1).

**Fig. 4:**
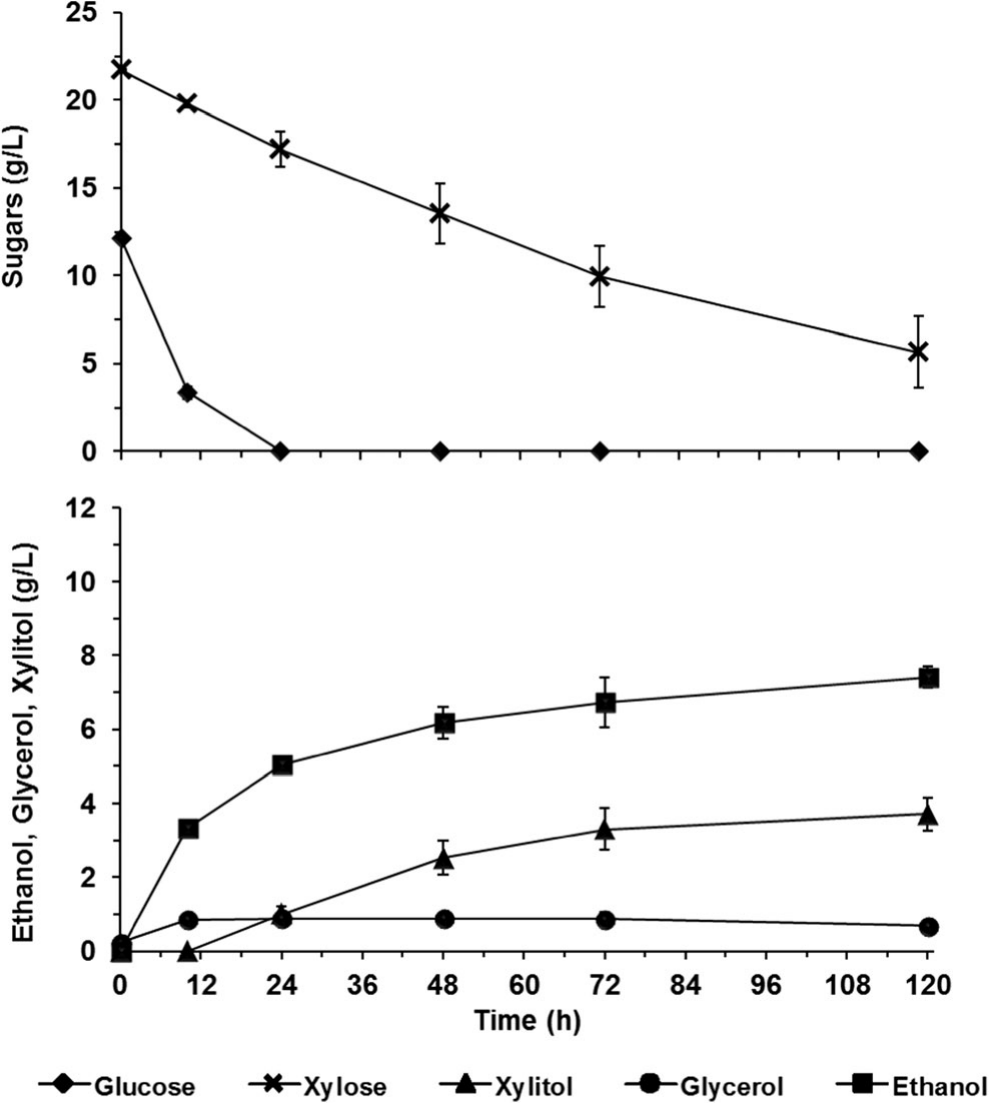
Fermentation of 50% (*v*/*v*) hydrolysate by the final evolved population *C. intermedia* EVO 2

After evolutionary engineering, the tolerance of the resulting populations to inhibitors was first evaluated by fermentation of 50% (*v*/*v*) hydrolysate. The evolved populations were able to ferment both glucose and xylose with similar fermentation profiles, in contrast to the parental strain (Fig. 4, Table 2, and Supplementary Fig. S2). Fermentation of 50% (*v*/*v*) hydrolysate for 72 h resulted in ethanol concentrations similar to those obtained during the fermentation of 40% (*v*/*v*) hydrolysate with the parental strain (6.7 g/L and 6.4 g/L for EVO 1 and EVO 2, respectively). These ethanol concentrations were further increased to 7.5 g/L after 120 h of fermentation, resulting in a final ethanol conversion yield of 0.26 g/g (Table 2). The higher ethanol concentrations were the result of the conversion of higher xylose concentrations by the engineered cells (Table 2). Together with ethanol, 1 g/L glycerol and 3.8 g/L xylitol were also accumulated (Fig. 4, Table 2, Supplementary Fig. S2). These results suggest that both the intermediate and the final evolved populations are more tolerant to lignocellulose-derived compounds than the parental strain, and thus better fit for converting xylose in the presence of inhibitors. Furthermore, it is indicative of the genetic stability of evolved populations, since EVO 2 was obtained in absence of lignocellulose-derived inhibitors.

**Fig. 5:**
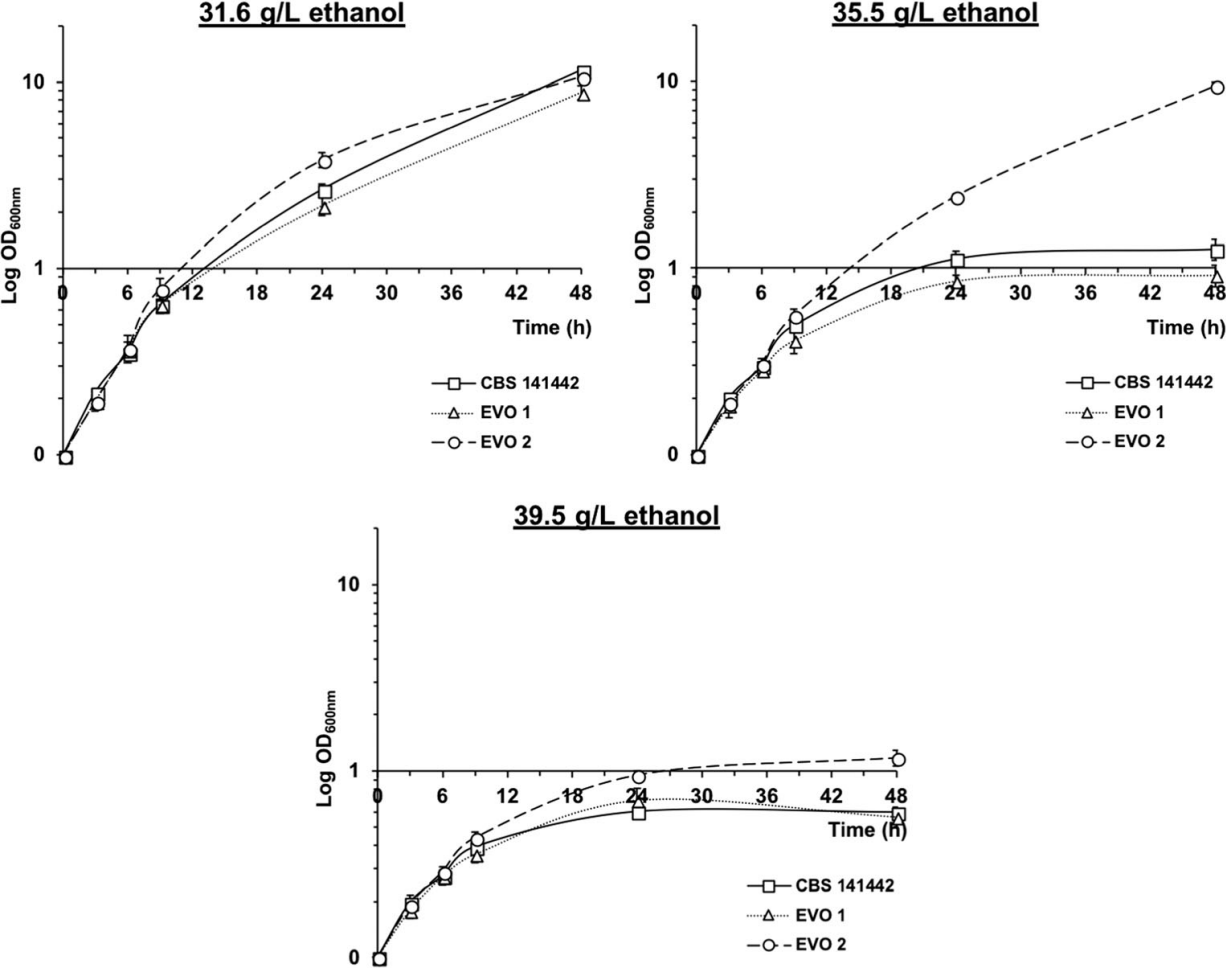
Biomass formation expressed as OD_600nm_ for the parental *C. intermedia* CBS 141442, and the evolved populations EVO 1 and EVO 2 after 48 h of cultivation in rich medium with xylose containing 31.6 g/L, 35.5 g/L, or 39.5 g/L ethanol

Finally, the tolerance of the evolved populations to ethanol was assessed by cultivation in YPX supplemented with 31.6 g/L, 35.5 g/L, or 39.5 g/L ethanol. These concentrations were found to be highly inhibitory to *C. intermedia* CBS 141442 (Fig. 3). At an ethanol concentration of 31.6 g/L, populations EVO 1 and EVO 2 showed similar growth patterns and final OD_600nm_ values to those observed for the parental strain (Fig. 5). However, an increase in the ethanol concentration from 31.6 to 35.5 g/L resulted in severe growth inhibition of the parental strain and the evolved population EVO 1, reaching a final OD_600nm_ of about 1, and showing no additional growth after 24 h of cultivation, probably due to energy exhaustion (Stanley et al. 2010). The same ethanol concentration (4.5% (*v*/*v*)) had a lower inhibitory effect on the EVO 2 population, which continued to grow for the full 48 h of measurements, reaching similar values of OD_600nm_ to those obtained with an ethanol concentration of 4% (*v*/*v*). However, a further increase in the ethanol concentration to 5% (*v*/*v*) had a strong inhibitory effect on EVO 2 cells, reaching a final OD_600nm_ slightly above 1 (Fig. 5). These results are indicative of the better ethanol tolerance of the final evolved population, and underline the importance of increasing microbial robustness for lignocellulosic bioethanol production.

## Discussion

In order to achieve cost-effective lignocellulosic bioethanol production, the fermenting microorganisms must convert glucose and xylose efficiently into ethanol in a highly challenging environment. Despite intensive efforts to develop xylose-fermenting strains of *S. cerevisiae*, the xylose uptake and the fermentation performance of this yeast have still not reached satisfactory levels (Moyses et al. 2016). The continuous study of native xylose-fermenting yeasts is therefore of the utmost importance to improve our understanding of xylose conversion and to identify key metabolic steps.

In this study, we have characterized the fermentation performance of the in-house isolated *C. intermedia* strain CBS 141442. Simultaneous co-fermentation of glucose and xylose was observed, although xylose was consumed at a lower rate than glucose. Glucose/xylose co-fermentation is a highly desirable trait in simultaneous saccharification and fermentation of lignocellulosic feedstocks, since this process configuration is characterized by high initial xylose concentrations (due to the solubilization of hemicelluloses during the pretreatment step), whereas glucose is continuously released during the fermentation process (Olsson et al. 2005). The preference for glucose over xylose in glucose/xylose mixtures has been observed for most yeast species studied, including *S. cerevisiae* and natural pentose-fermenting yeasts such as *S. stipitis*, *S. shehatae*, and also other *C. intermedia* strains (Panchal et al. 1988; Saito et al. 2017). In many cases, this is due to a repression mechanism that impedes the utilization of other carbon sources while glucose is available (Gancedo 1998). Gárdonyi et al. (2003) reported that *C. intermedia* exhibited strong inhibition of xylose utilization in the presence of glucose. Nonetheless, the results presented in the present paper suggest that *C. intermedia* CBS 141442 can assimilate xylose in the presence of glucose, which is a trait worth further exploration.

Moreover, microbial robustness to lignocellulosic inhibitors is of crucial importance for lignocellulosic bioethanol production. *C. intermedia* CBS 141442 proved to be more sensitive to lignocellulose-derived inhibitors in the xylose-fermenting phase than in the glucose-fermenting phase. Moreno et al. (2013) have also reported limited xylose consumption by the xylose-recombinant yeast *S. cerevisiae* strain F12 after glucose depletion. In their study, the low xylose consumption was found to be associated with a decrease in cell viability due to the stress exerted by the inhibitors on the yeast during fermentation. Similarly, *C. intermedia* CBS 141442 might suffer from a loss of cell viability during fermentation of 40% and 50% (*v*/*v*) hydrolysate due to exposure to high concentrations of lignocellulose-derived compounds. In spite of having inherent mechanisms for tolerating/converting some of the inhibitory compounds such as furfural and 5-HMF (these compounds can be converted into less inhibitory products by the fermentative microorganisms through oxidation and/or reduction processes), at a certain concentration the synergistic action of the cocktail of inhibitors in a hydrolysate becomes sufficiently high to stop fermentation completely. At the selected fermentation pH range of 5–5.5, acetic acid (pKa = 4.75) and formic acid (pKa = 3.75) present in the hydrolysate are predominantly in their non-protonated forms and thus expected not to dominate the toxicity effects on cells. However, steam-exploded wheat straw hydrolysates usually contain also phenolics and other compounds, not evaluated in this study, that might play significant roles and promote synergistic effects during the inhibition of *C. intermedia*.

Resulting ethanol concentrations must be sufficiently high (above 40 g/L) for the biomass conversion process to be economically viable. Ethanol concentrations of 23.7 g/L and 31.6 g/L severely affected the growth of *C. intermedia* CBS141442 in media containing glucose and xylose, while concentrations of 43–53 g/L were predicted to be completely inhibitory. Ethanol concentrations in the range of 5–7% (*v*/*v*) have also been reported to be completely inhibitory to species belonging to the *Hanseniaspora*, *Candida*, *Pichia*, *Kluyveromyces*, *Metschnikowia*, *Torulaspora*, and *Issatchenkia* genera (Ciani et al. 2016). In the case of *S. stipitis*, which is considered to be one of the best natural xylose-fermenting microbes, the maximum ethanol concentrations at which cells could not grow were estimated to be 33.6 g/L (glucose) and 44.7 g/L (xylose) (Lee et al. 2000), which are about 20% lower than those observed in the present study for *C. intermedia* CBS 141442 (Fig. 3c, d).

According to the results of the growth and fermentation tests performed, we can conclude that *C. intermedia* CBS 141442 has a modest tolerance to lignocellulose-derived compounds and ethanol, especially when utilizing xylose. Therefore, xylose conversion by this yeast strain might be improved by increasing its robustness to these compounds. Evolutionary engineering has proved to be a highly suitable method for enhancing traits in various microorganisms, including non-conventional yeast species for which we lack a detailed knowledge about their physiology as well as tools for targeted genome editing. Moreover, evolutionary engineering makes it possible to target complex polygenic phenotypes, such as tolerance to the cocktail of inhibitors present in lignocellulosic hydrolysates, without prior knowledge about the genes responsible for the trait (Steensels et al. 2014). For example, Nigam (2001) reported overcoming a 65-h lag phase in *S. stipitis* during fermentation of 60% (*v*/*v*) red oak acid hydrolysate through adaptation of cells on hardwood hemicellulose acid prehydrolysate, and the acetic acid tolerance of a *S. passalidarum* yeast strain was improved by an evolutionary engineering strategy based on UV-mutagenesis and subsequent selection by continuous cultivation (Morales et al. 2017). Similarly, the evolutionary engineering strategy employed in the present study resulted in the final evolved population *C. intermedia* EVO 2, which showed improved xylose-to-ethanol conversion in highly challenging environments. Here, the fermentation of 50% (*v*/*v*) hydrolysate, which was completely inhibitory to the parental stain, showed similar final ethanol concentrations and xylose consumption rates to those observed at lower inhibitor concentrations (30–40% (*v*/*v*) hydrolysate), independently of the evolved population used. This result suggests that stable genetic changes are responsible for the corresponding evolved phenotype, since population EVO 2 was obtained in absence of lignocellulose-derived inhibitors. Furthermore, the EVO 2 population showed improved ethanol tolerance when growing in xylose medium with an ethanol concentration of 35.5 g/L, a concentration that caused complete growth inhibition of CBS 141442 after 24 h. Although we cannot completely rule out the possibility that the increased ethanol tolerance is influenced by the rich medium used in the experimental setup, these results highlight the benefit of sequentially applying lignocellulose-derived compounds and ethanol as selective pressures during evolutionary engineering to produce strains with enhanced robustness to these inhibitory compounds. Future studies involve determining the underlying genetic changes responsible for the improved phenotypes, which can yield valuable insights into how the specific traits are established.

The native xylose-fermenting yeast *C. intermedia* has several traits of interest for the conversion of lignocellulosic biomass into bio-based products such as ethanol. These include rapid xylose uptake (Leandro et al. 2006), xylose reductases for better redox balance (Nidetzky et al. 2003), and the simultaneous utilization of both glucose and xylose during fermentation, as shown here. Furthermore, cells with enhanced robustness to lignocellulosic-derived inhibitors and ethanol were obtained by subjecting *C. intermedia* CBS141442 to evolutionary engineering. We believe that the findings of this study may contribute to our understanding of important physiological/genetic traits which will help in the further development and implementation of optimal lignocellulosic biomass conversion processes.

## Electronic supplementary material

ESM 1 (PDF 227 kb)
