# Supplementary material for: Evolutionary engineered Candida intermedia exhibits improved xylose utilization and robustness to lignocellulose-derived inhibitors and ethanol
Source: Appl Microbiol Biotechnol. 2018 Nov 29;103(3):1405–16. doi: 10.1007/s00253-018-9528-x (PMC6394480; doi:10.1007/s00253-018-9528-x)
Supplement: Supplementary file 1 — (PDF 227 kb) [file 253_2018_9528_MOESM1_ESM.pdf]

Applied Microbiology and Biotechnology

Evolutionary engineered *Candida intermedia* exhibits improved xylose utilization and robustness to lignocellulose-derived inhibitors and ethanol

Antonio D. Moreno[1,2], Antonella Carbone[1], Rosita Pavone[1], Lisbeth Olsson[1*] and Cecilia Geijer[1]

[1]*Chalmers University of Technology, Department of Biology and Biological Engineering, Division of Industrial Biotechnology, Gothenburg, Sweden*

[2]*CIEMAT, Department of Energy, Biofuels Unit, Madrid, Spain*

[*]Correspondence to: Lisbeth Olsson, Chalmers University of Technology, Department of Biology and Biological Engineering, Division of Industrial Biotechnology, SE-41296, Gothenburg, Sweden

E-mail: lisbeth.olsson@chalmers.se

Phone: + 46 31 772 38 05

Supplementary Information

Figure S1

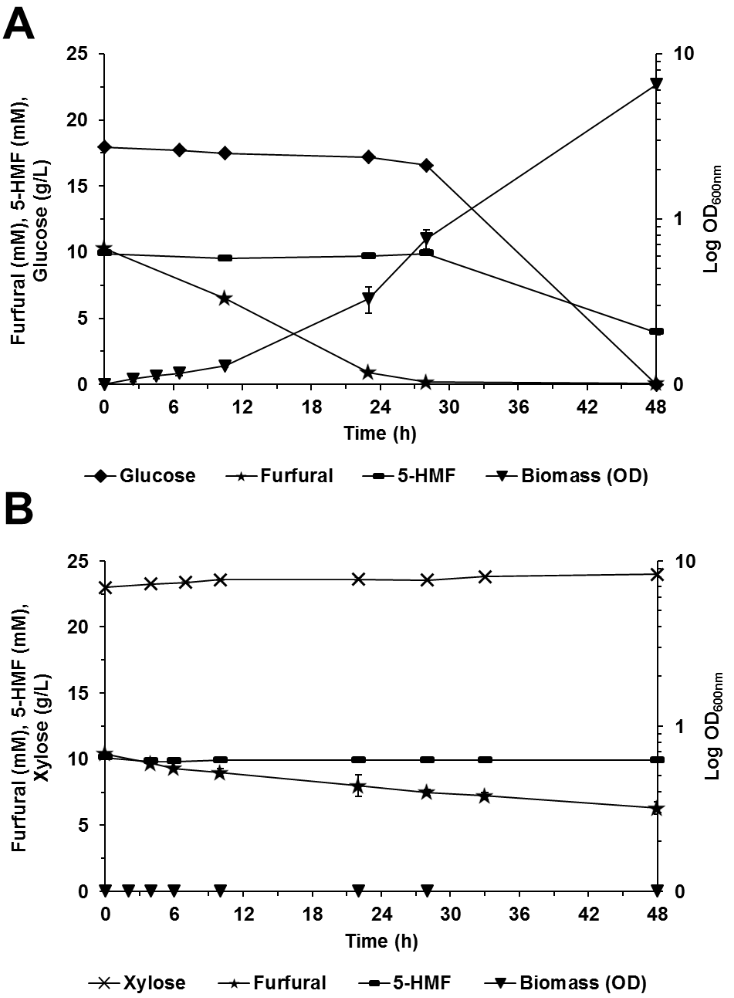

Figure S1. Effect of the furan derivatives furfural and 5-hydroxymethylfurfural (5-HMF) on *C. intermedia* strain CBS 141442 growing in mineral media with (**A**) glucose (MMD) and (**B**) xylose (MMX) as a sole carbon source.

<u>Methodology</u>

The ability of *C. intermedia* to tolerate and convert furfural and 5-hydroxymethylfurfural (5-HMF) was evaluated in MMD or MMX supplemented with 10 mM furfural (1.0 g/L) and 10 mM 5-HMF (1.3 g/L). Cells growing in the exponential phase in MMD or MMX were transfer to 50 mL of the corresponding test medium to a final OD_{600nm} of 0.1. Cultures were then incubated in an orbital shaker at 30 °C and 150 rpm for 48 h. Samples were withdrawn periodically to monitor the concentrations of sugars, furfural and 5-HMF.

Figure S2

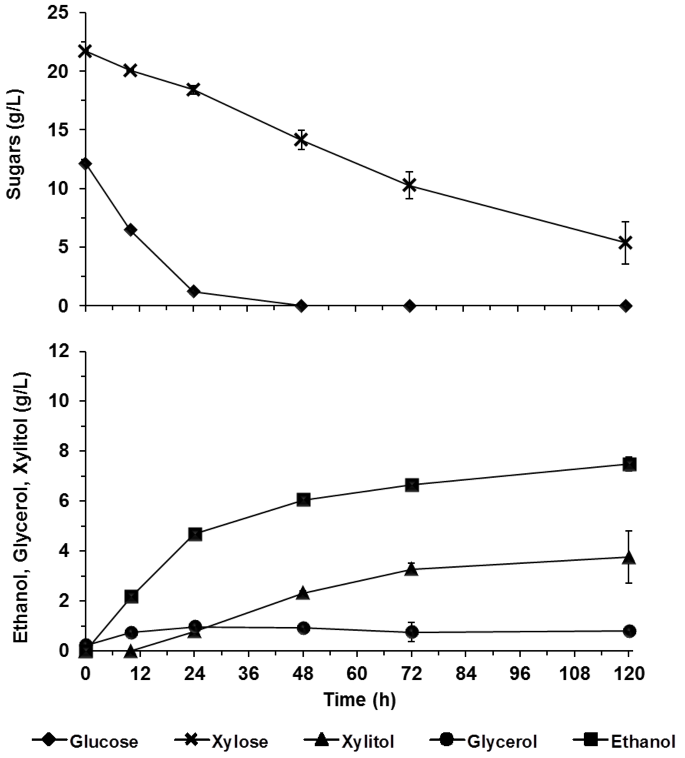

Figure S2. Fermentation of 50% (v/v) hydrolysate by the intermediate evolved population *C. intermedia* EVO 1.
